# Thymidylate synthase maintains the de-differentiated state of triple negative breast cancers

**DOI:** 10.1038/s41418-019-0289-6

**Published:** 2019-02-08

**Authors:** Aarif Siddiqui, Paradesi Naidu Gollavilli, Annemarie Schwab, Maria Eleni Vazakidou, Pelin G. Ersan, Mallika Ramakrishnan, Dick Pluim, Si’Ana Coggins, Ozge Saatci, Laura Annaratone, Jan HM Schellens, Baek Kim, Irfan Ahmed Asangani, Suhail Ahmed Kabeer Rasheed, Caterina Marchiò, Ozgur Sahin, Paolo Ceppi

**Affiliations:** 1 Junior Research Group 1, IZKF, FAU Erlangen-Nürnberg, Erlangen, Germany; 2 Department of Cancer Biology, Perelman School of Medicine, University of Pennsylvania, Philadelphia, PA USA; 3 Department of Molecular Biology and Genetics, Bilkent University, Ankara, Turkey; 4 Georg-Speyer-Haus Institute for Tumor Biology and Experimental Therapy, Frankfurt am Main, Germany; 5 Netherland Cancer Institute, Amsterdam, The Netherlands; 6 Department of Pediatrics, Emory University School of Medicine, Atlanta, GA USA; 7 Department of Drug Discovery and Biomedical Sciences, University of South Carolina, Columbia, SC USA; 8 Department of Medical Sciences, University of Turin, Turin, Italy; 9 Department of Pharmacy, Kyung Hee University, Seoul, South Korea; 10 Programme in Cancer and Stem Cell Biology, Duke-NUS Medical School, Singapore, Singapore; 11 Pathology Unit, Azienda Ospedaliera Universitaria Città della Salute e della Scienza di Torino, Turin, Italy; 12 Present Address: Pathology Unit, Candiolo Cancer Institute, FPO-IRCCS, Candiolo, Italy

**Keywords:** Cancer metabolism, Cancer stem cells

## Abstract

Cancer cells frequently boost nucleotide metabolism (NM) to support their increased proliferation, but the consequences of elevated NM on tumor de-differentiation are mostly unexplored. Here, we identified a role for thymidylate synthase (TS), a NM enzyme and established drug target, in cancer cell de-differentiation and investigated its clinical significance in breast cancer (BC). In vitro, TS knockdown increased the population of CD24^+^ differentiated cells, and attenuated migration and sphere-formation. RNA-seq profiling indicated repression of epithelial-to-mesenchymal transition (EMT) signature genes upon TS knockdown, and TS-deficient cells showed an increased ability to invade and metastasize in vivo, consistent with the occurrence of a partial EMT phenotype. Mechanistically, TS enzymatic activity was found essential for maintenance of the EMT/stem-like state by fueling a dihydropyrimidine dehydrogenase—dependent pyrimidine catabolism. In patient tissues, TS levels were found significantly higher in poorly differentiated and in triple negative BC, and strongly correlated with worse prognosis. The present study provides the rationale to study in-depth the role of NM at the crossroads of proliferation and differentiation, and depicts new avenues for the design of novel drug combinations for the treatment of BC.

## Introduction

Tumor de-differentiation contributes to the malignant phenotype of most solid tumors, including breast cancer (BC) [1, 2], and in many cases this is achieved through the epithelial-to-mesenchymal transition (EMT) and the cancer stem cell (CSC) programs [3–5]. Indeed, EMT and CSC markers are more frequently found in aggressive poorly differentiated (high-grade) tumors in breast [2] and other cancers [6–10]. Understanding the mechanisms controlling the differentiation of BC cells can therefore lead to more effective therapeutics.

Nucleotide metabolism (NM) is classically viewed as a motor of cellular proliferation [11]. Cancer cells are, in fact, highly dependent on the de novo synthesis of nucleotides to produce sufficient DNA and RNA precursors to support their growth, and some cancer-promoting signaling pathways have been shown to regulate NM [12]. However, a few recent studies have suggested that nucleotides-generating metabolic pathways may also serve as regulators of cancer stemness [13, 14], opening the possibility that some NM enzymes are implicated in cancer cell de-differentiation.

Thymidylate synthase (TS) is the enzyme that catalyzes the conversion of deoxyuridine monophosphate (dUMP) to thymidine monophosphate (dTMP or thymidylate). Since this reaction provides the sole de novo pathway for thymidylate production, TS is essential for DNA synthesis and repair, and its absence blocks proliferation and causes cell death [15, 16]. We previously discovered that TS expression is correlated with the EMT phenotype in the NCI-60 transcriptomic database by using a pan-cancer EMT gene ratio (Vimentin/E-Cadherin, VIM/CDH1) [17], but the mechanistic involvement of the TS enzymatic activity on EMT/CSCs has never been shown. Here, we report a novel fundamental role of TS in maintaining the de-differentiated phenotype of BC cells and its differential expression in the BC subtypes, with several potential therapeutic implications.

## Material and methods

### Cell lines

MDA-MB-231 (ATCC), BT-549 and T-47D (NCI) were cultured in RMPI-1640 (Sigma), while Hs 578T (DSMZ) were cultured in DMEM (Sigma). Media were supplemented with 10% FBS (Sigma), 1%Pen/Strep (Sigma) and 1%L-Glutamine (Sigma). Cells were STR-profiled, used between passages 3 and 15, examined for mycoplasma and maintained in Plasmocin (Invivogen) to prevent contamination. Human umbilical vein endothelial cells (HUVEC) were cultured in Ham’s F-12K Medium (Gibco) supplemented with 0.1 mg/ml heparin (Sigma) and 0.03 mg/ml endothelial cell growth supplement (Sigma) 50 μ/ml Pen/Strep (Lonza) and 10% FBS (Biowest).

### Western blot analysis

Cells were lysed in RIPA buffer and quantified using the Pierce BCA kit (Thermo-Fisher). Proteins lysates (10–35 μg) were resolved on 10% SDS–PAGE gels and transferred to PVDF membrane (Thermo-Fisher). Membranes were blocked in 5% Milk (BioRad) in 1XTBS-T and incubated overnight in primary antibodies diluted in 5% milk at 4 °C. Western blot antibodies for TS (EPR4545) and DPYD (EPR8811) are from Abcam and β-Actin (8H10D10) is from Cell Signaling. After incubation with secondary antibodies (Southern Biotech), detection was performed using the ECL (Thermo-Fisher) and developed on X-Ray film (Thermo-Fisher) using a chemiluminescence imager, AGFA CP100.

### Proliferation assay

For proliferation assay cells were seeded in 96-well plates in low density (5–20% initial confluency). Plates were loaded in  IncuCyte ZOOM (Essen BioScience) and scanned every 2–4 h. For each scan, phase contrast image was acquired from every well and was analyzed by IncuCyte Zoom software.

### Migration assay

For migration assay cells were plated in 96-well plates so that they reach 90% confluency overnight. Cells were wounded using WoundMaker (Essen BioScience) as per the instruction from the manufacturer. Plates were loaded in IncuCyte ZOOM and were automatically scanned for programmed time interval. For each scan, wound width was recorded by the software and the proliferation inside the wound was normalized to the proliferation outside the wound, giving relative wound density for each time point.

### Cell surface staining and FACS

Anti-CD44-FITC and anti-CD24-PE antibodies are from Biolegend. For staining, 400,000 cells were plated in 6-well plate and allowed to grow overnight. Cells were trypsinized and collected in FACS tubes. After two washes with 1% FBS/PBS, cells were incubated with 400 ng CD24-PE and CD44-FITC (diluted in 100 µl 1% FBS/PBS) for 20 min on ice. Cells were washed twice with 1% FBS/PBS and suspended in 5 mM EDTA and 2% FBS/PBS. CD24/CD44 positivity was recorded in CytoFLEX flow-cytometer (Beckman Coulter). Gating was done using unstained cells. FACS data were analyzed using FlowJo (V10.1).

### RNA sequencing

Total RNA was extracted using miRNeasy kit (Qiagen) following the manufacturer’s instructions. RNA-Seq libraries were constructed using the TruSeq sample Prep Kit V2 (Illumina). Briefly, 1 μg of purified RNA was poly-A selected and fragmented with fragmentation enzyme. After first and second strand synthesis from a template of poly-A selected/fragmented RNA, other procedures from end-repair to PCR amplification were done according to library construction steps. Libraries were purified and validated for appropriate size on a 2100 Bioanalyzer High Sensitivity DNA chip (Agilent Technologies.). The DNA library was quantified using Qubit and normalized to 4 nM before pooling. Libraries were pooled in an equimolar fashion and diluted to 10 pM. Library pools were clustered and run on Nextseq500 platform with paired-end reads of 75 bases, according to the manufacturer’s recommended protocol (Illumina). Raw reads passing the Illumina RTA quality filter were pre-processed using FASTQC for sequencing base quality control. Sequence reads were mapped to UCSC human genome build using TopHat and differential gene expression determined using Cufflinks 2.1.1 and Cuffdiff2.1.1 as implemented in BaseSpace. Sequencing data has been deposited to publically available GEO dataset GSE122953.

### Bioinformatic analysis

Gene set enrichment analysis (GSEA) on the differentially-expressed genes (DEGs) upon TS knockdown was performed with the Molecular Signatures Database v6.1 software. For patient data analysis, normalized gene expression data from the following patient datasets were downloaded from GEO database; GSE19783, GSE31448, GSE19536, GSE58644, GSE21653, GSE45827, and GSE58812. While GSE58812 is a dataset of triple-negative BC patients, all other datasets are of BC patients with different subtypes. For calculation of TS Knockdown (KD) score, first, *z* scores of the down- and up-regulated genes upon TS knockdown were calculated. Then, the sum of *z* scores of downregulated genes were subtracted from the sum of *z* scores of upregulated genes and KD scores were obtained for each patient. Patients were grouped based on either their TYMS gene expression or KD score. GSEA was performed by using patient data from GSE58644 and GSE58812. Survival graphs were generated in GraphPad and the significance was assessed by Log-rank test. Survival graphs from the KM Plotter database was generated based on TYMS expression by using the auto select best cutoff option. Statistical analyses were performed by unpaired student’s *t*-test.

### Quantitative real-time PCR

Total RNA was extracted using miRNeasy kit (Qiagen) and converted to cDNA using Tetro cDNA synthesis kit (Bioline) with random hexamers. 50 nanograms cDNA was used as template for real-time quantification. GAPDH was used as the internal control. TaqMan probes (Thermo-Fisher) were used for quantification in Applied Biosystems 7300. Fold change was calculated using the ΔΔCt method.

### Mouse tail vein metastasis injection

For tail vein metastasis assay, 1.5 × 10^6^ cells were injected into tail vein of 6–8 weeks old female athymic nu/nu mice, with three mice per group. Lung metastases were monitored by bioluminescence imaging (BLI). Anesthetized mice were intraperitoneally injected with 200 mg/kg D-luciferin (Perkin Elmer). Bioluminescence images were acquired with Lumina III in vivo Imaging System (Perkin Elmer). Analysis was performed with live imaging software by measuring photon flux. Lungs were collected and fixed in 10% buffered formalin and processed to obtain paraffin blocks. Three-micron thick sections of formalin fixed paraffin embedded samples were stained using hematoxylin and eosin. Mice experiments were approved by Animal Ethics Committee of the Bilkent University.

### Real-time HUVEC invasion assay (in vitro extravasation assay)

In vitro extravasation assay was performed as previously described [18]. Briefly, 2.5 × 10^4^ HUVEC cells in 100 µl were seeded in E-plates (ACEA xCelligence). Once HUVEC monolayer formed (19–21 h), 1 × 10^4^ MDA-MB-231 cells were added on top of the monolayer. A decrease in cell index indicates invasion through the HUVEC monolayer by tumor cells. Penetration of HUVEC monolayer was monitored for up to 10 h by using xCelligence Real-time Cell Analyzer (Acea Biosciences).

### Chick chorioallantoic membrane (CAM) assay

CAM assay for intravasation was performed as described previously [19]. Briefly, fertilized white leghorn eggs (Asby) were incubated in a rotary incubator at 37 ^o^C with 60% humidity for 10 days. The CAM was dropped by drilling a small hole through the eggshell into the air sac and a second hole near the allantoic vein that penetrates the eggshell membrane but not the CAM. The CAM was dropped by applying a mild vacuum to the hole over the air sac. Subsequently, a cutoff wheel (Dremel) was used to cut a 1 cm^2^ window, encompassing the second hole near the allantoic vein to expose the underlying CAM. The CAM was gently abraded with a sterile cotton swab to provide access to the mesenchyme and 50 μl inoculum of 2 × 10^6^ MDA-MB-231-pLKO (*n* = 10) or MDA-MB-231-shRNA-TS#2 (*n* = 10) cells were applied. Four eggs without human cells are used as negative control. The windows were subsequently sealed and the eggs returned to incubator. The eggs remained in the incubator for further 2 days, following which the CAM was cut into two halves and the tumor was excised from the upper CAM and the genomic DNA was extracted from the lower CAM. The lower CAM genomic DNA was then analyzed for the presence of human cells by quantitative *Alu* PCR with 500 ng cDNA. The number of intravasated human cells was then plotted in the graph as shown.

### Deoxynucleotide triphosphate quantification

The cellular dNTP levels were determined by the RT-based dNTP assay [20]. Briefly, the cellular dNTPs in experimental triplicates were extracted by methanol, and the determined dNTP amounts were normalized for an equal cell number (1 × 10^6^).

### TS enzyme activity quantification

TS was quantified in MDA-MB-231 cells as previously described [21]. Briefly, cells were collected and pelleted. Cells were suspended in 300 µl ice-cold Reaction mix (RM, 20 mM MgCl_2_, 1.5 mM NaF, 1 mM DTT in 50 mM Tris-HCl pH 7.5, after deoxygenation 0.47 (v/v%) BME was added. Next, cell lysates were prepared on ice by applying 15 pulses with a Branson 250 tip sonicator (Branson) at power input setting level 3 with a 50% duty cycle. After centrifugation at 11000 g for 20 min at 4 °C, 95 µl of supernatant was transferred to a clean 1.5 ml vial on ice for immediate determination of protein followed by TS activity analysis. Protein concentrations in PBMC cytosolic lysates were determined using the Bio-Rad protein assay (Bio-Rad). Briefly, 5 µl of PBMC cytosolic lysate was diluted with 45 µl of MilliQ water (Millipore). Five bovine serum albumin standards were prepared in concentrations ranging from 32.5 to 500 mg/ml to obtain a standard curve. In duplicate 10 µl of diluted lysate and the standard curve were transferred to a clear 96-well flat bottom plate. After the addition of 200 µl dye solution, the plate was incubated for 15 min at RT and subsequently the absorption was measured at 590 nm using an EL340 microplate reader (Bio-Tek). Immediately before the start of TS activity assay, a vial containing 2.51 mg of lyophilized MTHF was reconstituted in 500 µl of deoxygenized water and 10 µl was added to a 2.0 ml vial on ice. To this vial 85 µl of ice-cold tumor cell cytosolic lysate corresponding to 15 µg of protein was added. Next, 5 µl of 1 mM ice-cold substrate was added, and after mixing, the samples were incubated for 3 h at 37 °C in a shaking water bath. The reaction was terminated by adding 100 µl of 6.5 N HCl, and the remaining substrate was bound onto 400 µl Carbon slurry (CS, 5 g acid washed charcoal, 50 mg Dextran T500 in phosphate buffered saline) by vertical disk rotation mixing of the samples at 50 rpm at 4^o^ C. After centrifugation at 11,000 g for 5 min at 4 °C, 300 µl of clear supernatant was transferred to a 20 ml polyethylene vial, mixed with 10 ml of Ultima Gold, and assayed for radioactivity for 5 min using a LSC2900 Tri-Carb liquid scintillation counter.

### Lentiviral transduction

Plasmids for TS (TRCN000045663/66/67) and DPYD knockdown (TRCN000025799) are from Sigma. Empty vector (pLKO) was used as control. For TS reconstitution, plasmids were purchased from GeneCopoeia in which silent mutation was introduced in shTS#1 binding region (5′-G_483_CAAAGAGTAATCGATACAAT_503_–3′). Enzymatically inactive TS expression vector was generated by introducing single point mutation (5′-C_148_GC_150_–3′→5′-TGC-3′) in reconstitution vector. Empty vector (CS-T0406-LV151) was used as control. TS expression vector (Ex-T0406-LV105b) and control vector (Ex-Neg-LV105b) are from GeneCopoeia. For production of lentiviral particles, 293T cells were transfected with 8 µg knock-down/expression vectors and 2 µg of pMDL, pV_s_V_g_ and pRevRes in complex with 24 µg PEI (Polysciences). After 48 h, supernatant was collected, centrifuged and filtered. For transduction, 100,000–150,000 cells were seeded in 6-well plate and infected presence of 8 μg/ml polybrene (Sigma). Selection was done with 3 μg/ml puromycin (Sigma) and cells were maintained in 1 µg/ml puromycin. MDA-MB–231 cells infected with TS reconstitution vectors were selected in 800 µg/ml G418 (Sigma) and cultured in 250 µg/ml G418.

### Mammosphere culture

Forty thousand cells were seeded in triplicates in ultra-low attachment 6-well plates (Corning) in complete Mammocult medium (Stem Cell Technologies), prepared according to the manufacturer’s instruction. After formation, spheres were counted by spinning at 300 g for 5 min and suspending in PBS (Lonza).

### In vitro cell treatment

Pemetrexed was purchased from Sigma, dihydrothymine (DHT) was purchased from Selleckchem and were dissolved in DMSO to final concentration of 100 mM and 40 mM respectively. Cisplatin was purchased from Santa Cruz Biotechnology and was dissolved in PBS to the final concentration of 80 mM. For in vitro treatment cells were plated in 96-well plates (MDA-MB-231 = 3000–4000 cells/well and T-47D = 6000 cells/well) and incubated overnight. For cytotoxicity death assay, 2000X Cytotox Green Reagent (Essen BioScience) was diluted in RPMI and working dilutions of pemetrexed and cisplatin were prepared in Cytotox Green supplemented media. Working dilutions of DHT were prepared in RPMI (without Cytotox Green). After treatment, plate was loaded in Incycuyte Zoom and images were acquired in real-time for phase to quantify growth. Activity of Cytotox reagent was simultaneously acquired at the green channel to quantify death. Incycuyte Zoom software was used for the analysis and data export.

### Patients and samples

Formalin-fixed paraffin embedded samples of a series of 120 consecutive breast carcinomas collected between 2010 and 2013 at the Azienda Ospedaliera Universitaria Città della Salute e della Scienza di Torino were analyzed. All cases were reviewed and anonymized using an alpha-numerical code. The main patients’ characteristics are shown in Supplementary Table 2. The use of retrospective solid tumor tissues for the immunohistochemical study was approved by the Ethic Institutional Review Board responsible for “Biobanking and use of human tissues for experimental studies” - Department of Medical Sciences, University of Turin.

### Immunohistochemistry staining and scoring

Three micrometer thick sections of formalin fixed paraffin embedded samples were stained for immunohistochemistry with the EPR4545 antibody (Abcam, 1:150 dilution). Immunohistochemistry was performed using an automated slide-processing platform (Ventana BenchMarck XT Autostainer). The antibody was optimized on FFPE cell block sections of MDA-MB-231 cells and on human tonsil tissue. Positive and negative controls were included for each immunohistochemical run. Immunoreactivity was assigned based on the proportion of positive tumor cells over total tumor cells (percent positivity) ranging from 0 to 100%. Staining intensity was evaluated as negative, faint, moderate, and intense. If the staining intensity was heterogeneous, then scoring was based on the greatest degree of intensity. Data were integrated in the *H*-score, calculated as follows: *H*-score = ΣPi (i + 1), where *i* represents the intensity of staining (0–3+), and Pi stands for the percentage of stained tumor cells (0–100%).

### Statistical analysis

Statistical tests were performed with the GraphPad software v.7 comparing groups of different conditions with replicates. In all tests, the statistical significance was set at *p* = 0.05 (in the figures * indicates *p* < 0.05, **p < 0.01, ***p < 0.001).

## Results

### TS expression correlates with aggressive form of BC

**Fig. 1:**
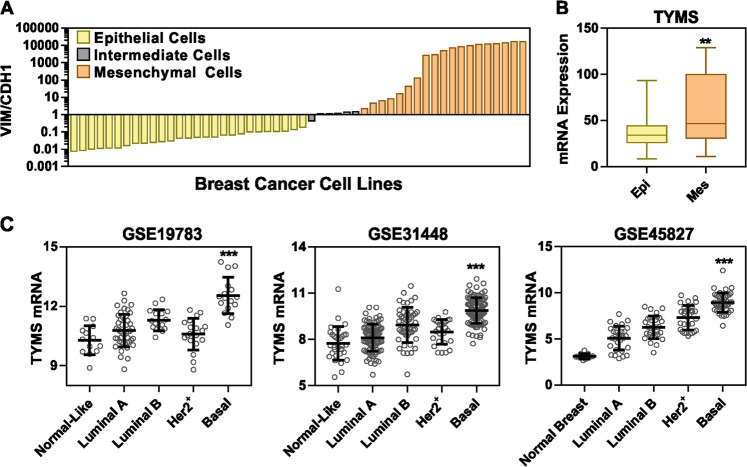
TS expression correlates with aggressive form of BC. **a** Vimentin/E-cadherin (VIM/CDH1) ration in BC cell lines belonging to the CCLE database (gene expression). Cells were sorted in epithelial (*n* = 27), EMT-intermediate (*n* = 6) and mesenchymal (*n* = 19) phenotype. **b** Comparison of TS mRNA expression levels between epithelial and mesenchymal BC cell lines (*p* = 0.0039, two-tailed *t*-test). **c** TS mRNA levels in BC tissues divided according to molecular subtypes in indicated GEO datasets (*p* < 0.0001 in all datasets, compared between luminal A and basal subtypes, one-way ANOVA, Turkey’s multiple comparison)

In order to study the association of TS expression with EMT markers in BC, we employed a VIM/CDH1 ratio to classify the BC cell lines belonging to the CCLE dataset (*n* = 52) into epithelial, mesenchymal or intermediate phenotypes (Fig. 1a and Supplementary Table 1. Comparing epithelial (VIM/CDH1 < 2) with mesenchymal cells (>2), a significantly higher expression of TYMS mRNA was found in the latter (*p* < 0.005, Fig. 1b). Then, in order to test TS gene expression in the spectrum of BC patients, we analyzed three independent GEO datasets, and found that TS expression was significantly different among the BC subtypes. Normal-like samples or the well-differentiated tumors (like luminal A) exhibited low TS expression, whereas high TS levels were found in basal-like BC (Fig. 1c). BC with a basal-like gene signature are primarily triple-negative (TN), and are frequently enriched for and EMT markers [22].

### TS is a marker of more aggressive, chemoresistant, and EMT-driven BC

**Fig. 2:**
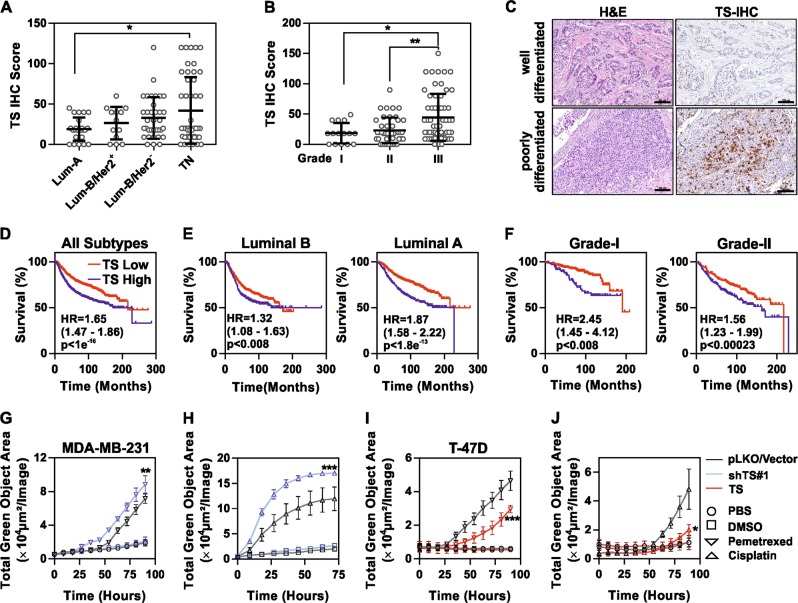
TS is a marker of aggressive, chemoresistant and EMT-driven BC. **a** Immunohistochemical staining of TS in FFPE samples from BC patients (*n* = 120) segregated according to molecular subtype or **b** grade of differentiation (*p* = 0.0237 between luminal A and TNBC and 0.0119 between Grade I and III, one way ANOVA, Turkey’s multiple comparison). Grade III are poorly differentiated cancers. **c** Representative images showing H&E and TS staining in well-differentiated and poorly-differentiated tumors (scale bars represent 100 µm). Kaplan–Meier curves showing the prognostic significance of TS expression in BC (all types) (**d**), in luminal (**e**), and in low grade patients (**f**). *P-*values are log-rank tests and HR is hazard ratio. MDA-MB-231 cells with shTS#1 knockdown, treated with (**g**) 1.5 mM pemetrexed and (**h**) 500 µM cisplatin (*p* = 0.0019 for pemetrexed and <0.0001 for cisplatin, between treatment of shTS#1 and pLKO, two way ANOVA, Turkey’s multiple comparison). Death is recorded as green objects that represent the dying cells that incorporate Cytotoxic Green dye. T-47D cells overexpressing TS (TS, red) and empty vector (Vector, black) treated with 1.5 mM pemetrexed (**i**) and 50 µM cisplatin (**j**) in presence of Cytotoxic Green dye (*p* = 0.0009 for pemetrexed and 0.0341 for cisplatin, between treatment of empty vector and TS, two-way ANOVA, Turkey’s multiple comparison). Experimental data are representative of at least two independent experiments with similar results. Points are avg ± SD

In order to test the clinical significance of the TS protein and its association with the aggressive phenotype in BC, immunohistochemistry (IHC) was performed on formalin-fixed paraffin-embedded samples from 120 BC patients. Patients’ characteristics are shown in the Supplementary Table 2. TS staining was quantified by an IHC score and significantly higher expression was found in the TNBC, as compared to Luminal-A (Fig. 2a) and in high grade (G3) compared to low grade tumors (Fig. 2b, c). A moderate level of correlation was found between TS and Ki67 proliferation marker (*R*_s_ = 0.48, Supplementary Fig 1A). Analysis of the prognostic values using large patient datasets indicated TS expression as a marker for poor overall survival in BC (all subtypes, Fig. 2d). Of note, TS prognostically stratified luminal A and luminal B patients (Fig. 2e) as well as lower grade patients (Fig. 2f), while no association was not found in more aggressive BC (Supplementary Fig 1B).

Moreover, since TS is a common anti-cancer drug target, being required for DNA repair and directly inhibited by compounds like pyrimidine or folate analogues [23], we additionally tested if change in TS expression altered BC cells’ sensitivity to the anti-folate drug pemetrexed and to cisplatin, in a similar fashion as previously observed in NSCLC [19] [24]. As a result, we found that MDA-MB-231 cells were sensitized to in vitro drug treatment after TS knockdown (Fig. 2g, h), whereas TS-low T-47D cells, overexpressing ectopic TS, showed increased resistance (Fig. 2i, j).

### TS is essential for the maintenance of a de-differentiated stem-like state in BC

**Fig. 3:**
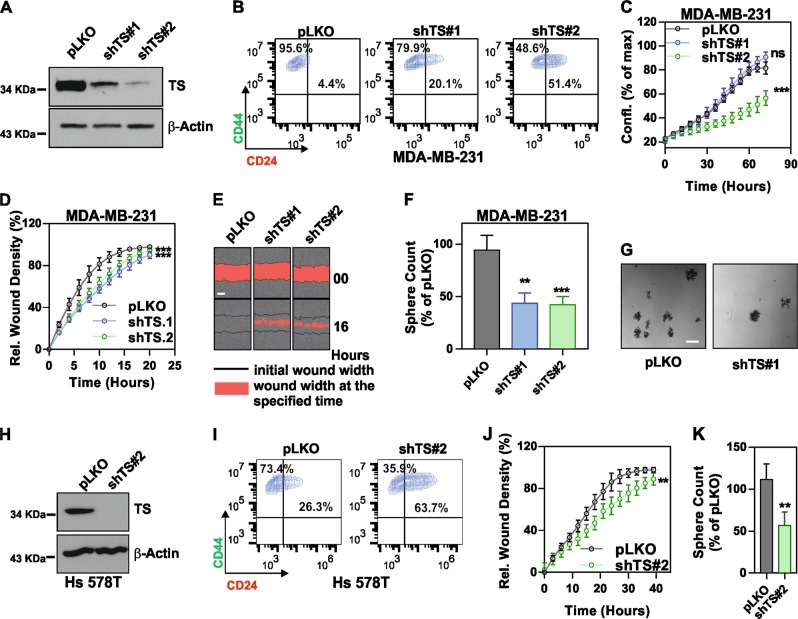
TS knockdown suppresses the mesenchymal phenotype of TNBCs. **a** Efficiency of TS knockdown in MDA-MB-231 obtained with shRNA lentiviral particles. pLKO is non-targeting control. **b** FACS plots of MDA-MB-231 cells as in **a** stained with CD44-FITC and CD24-PE. Gates are based on unstained control cells. **c** Real-time measurement of growth (confluency) in MDA-MB-231 with shTS or control cell (*p* = ns for shTS#1 and <0.0001 for shTS#2, as compared to pLKO, two-way ANOVA, Dunett’s multiple comparison). **d** Real-time migration assay in MDA-MB-231 cells with TS knockdown (*p* < 0.001 for shTS#1 and shTS#2, as compared to pLKO, two-way ANOVA, Dunett’s multiple comparison) and **e** representative pictures showing wound width at different time points (scale bar represents 300 µm). **f** Relative counts of spheres (*p* = 0.0012 for shTS#1 and 0.0001 for shTS#2 as compared to pLKO, one way ANOVA, Dunett’s multiple comparison) and **g** representative pictures of spheres formed by TS knockdown cells (scale bar represents 400 µm). **h** Efficiency of TS knockdown in Hs 578T obtained with shRNA lentiviral particles. **i** FACS plot of shTS and pLKO cells showing the expression of cell surface CD24/CD44. **j** Effect of TS knockdown on migration of Hs 578T cells (*p* = 0.0016, two-way ANOVA, Sidak’s multiple comparison). **k** Relative counts of spheres in Hs 578T TS knockdown cells as compared to pLKO (*p* = 0.0011, unpaired Student’s *t*-test). Experimental data are representative of at least two independent experiments with similar results. Points are avg ± SD

The patient data prompted us to test whether TS plays direct role in maintaining the de-differentiated state of TNBC. Hence, we transduced the TNBC cell line MDA-MB-231 with lentiviruses containing non-overlapping shRNA sequences to knockdown TS (Fig. 3a). CD44/CD24 surface staining followed by FACS quantification indicated an increase in the population of differentiated CD24^+^ cells, with a concomitant decrease in the CD44^+^CD24^−^ BC stem cell population (Fig. 3b). In order to carefully monitor the effects on cell growth, the confluency of infected cells was examined with real-time proliferation assays after TS depletion. The results showed a significant suppression of proliferation in cells infected with the shRNA that delivered a strong TS repression (shTS#2), while the sequence with a mild knockdown (shTS#1) did not alter the cells’ growth (Fig. 3c). By contrast, a shRNA (shTS#3) sequence that induced a complete TS elimination in this cell line caused a massive growth arrest and death (Supplementary Fig. 2A, B), in line with the life-essential role of TS. To determine if the partially TS-deficient cells had reduced ability to migrate, we performed a real-time wound-healing assay where cell migration is corrected for proliferation outside the wound to account for changes in cell growth rate upon TS repression. The results showed a significant loss of migratory ability upon TS knockdown (Fig. 3d, e). Moreover, assayed for stemness-related functional phenotype, TS-deficient cells formed less mammospheres in low-adherent cultures (Fig. 3f, g). Comparable findings were obtained in other TNBC cell lines, such as Hs 578T (Fig. 3h–k) and BT-549 (Supplementary Fig. 3A–D), implying that TS could be a common EMT/CSCs regulator in TNBC.

**Fig. 4:**
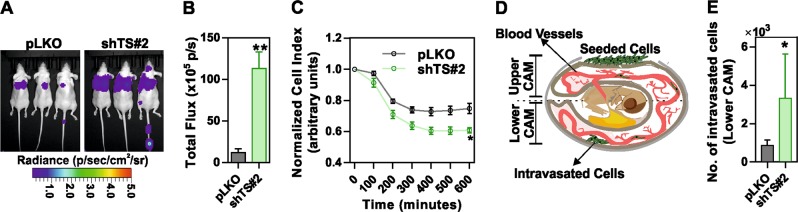
Ablation of TS expression alters the invasive ability of TNBC cells. **a** Representative images of lung metastasis formation assay performed by injecting luciferase-expressing MDA-MB-231 cells in the tail-vein of nude mice (*n* = 3/group). **b** Quantification of the bioluminescence signal from the lung metastases (*p* = 0.0078, unpaired two tailed *t*-test). **c** Relative ability of TS knockdown or control cells to penetrate through a HUVEC monolayer in an in vitro extravasation assay (*p* = 0.00065 unpaired multiple *t*-test). **d** Sketch diagram representing the chorioallantoic membrane (CAM) assay and **e** quantification of control and MDA-MB-231 shTS#2 intravasation in lower CAM, 2 days after seeding in the upper CAM (*p* = 0.0012, multiple *t*-test). Experimental data are representative from at least two independent experiments with similar results. Points are avg ± SD

Finally, to test the in vivo effects of TS suppression, MDA-MB-231-luc cells with TS knockdown or non-targeting control cells were injected in the tail-vein of nude mice. Interestingly, a significantly higher luciferase signal was recorded from shTS cells compared to control cells (Fig. 4a, b). Histological analysis confirmed the presence of single or multiple metastatic deposits of various size (ranging from <1 to 2 mm) and signs of lympho-vascular invasion only in mice injected with shTS cells (not shown). The increased ability of TS-deficient cells to form metastasis was independently confirmed by an in vitro real-time extravasation assay, which monitors how fast the cancer cells penetrate a monolayer of human umbilical vein endothelial cells (HUVEC) (Fig. 4c). In addition, to monitor the intravasation ability, a CAM assay on chicken embryos was performed (Fig. 4d), which confirmed an increased ability of TS-deficient cells, seeded on the upper CAM, to intravasate into the lower CAM (Fig. 4e). These observations clearly pointed at a strong impact of TS on BC differentiation and alteration of cells’ behavior, which we aimed to further molecularly characterize.

### TS knockdown induces loss of EMT and correlates with less aggressive BC

**Fig. 5:**
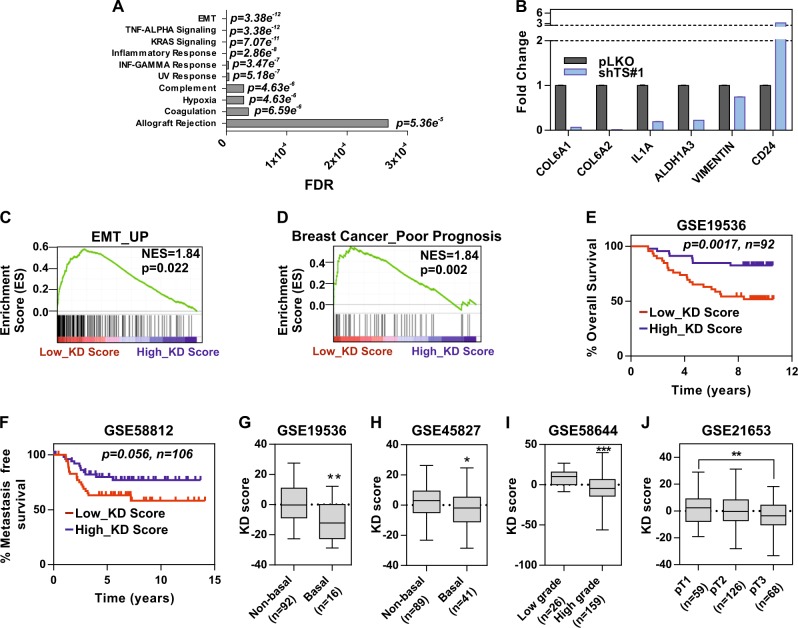
TS knockdown suppresses EMT and the associated gene signature correlates with less aggressive BC. **a** Gene set enrichment (GSEA) analysis showing the most differentially regulated pathways in MDA-MB-231 with TS knockdown (RNAseq data). **b** qPCR validation of RNA-seq results in MDA-MB-231 shTS#1. Bars are avg ± SEM. GSEA of TS low and high TS KD score (derived from RNA-seq) with previously published (**c**) EMT and **d** BC poor prognosis gene signatures, representing NSE (Normalized Enrichment Score) and *p*-value. Kaplan-Meier curves of **e** overall survival and **f** metastasis-free survival in cancer patients stratified by TS KD score. *P-*values are log-rank tests. TS KD scores in BC patients divided by **g**–**h** molecular subtype (*p* = 0.0022 for GSE19536 and 0.0333 for GSE45827, unpaired two tailed *t*-test), **i** grade of differentiation (*p* < 0.0001, unpaired two tailed *t*-test), and **j** TNM tumor size (*p* = 0.0091, one-way ANOVA, Turkey’s multiple comparison). Experimental data are representative from at least two independent experiments with similar results

To further delineate the molecular pathways regulated via TS and involved in mediating the de-differentiation program in BC, we subjected shTS#1 MDA-MB-231 cells to RNA-sequencing. These cells were selected because they had a discernable change in the CD24^+^ population but no major proliferation defect, so to analyze proliferation-independent effects. By setting a stringent cutoff value of 2-fold to identify DEGs compared to non-targeting infected cells (pLKO), we found 73 and 84 genes down and up-regulated, respectively (Supplementary Table 3). Pathway analysis (GSEA) revealed that EMT was the most significantly deregulated pathway, followed by TNF-α/NFκ-β signaling (Fig. 5a), known to be functionally connected with EMT in BC [25]. RNA-seq data were validated by qPCR (Fig. 5b, Supplementary Fig. 4A) and were used to explore the prognostic significance of the gene signature associated with TS-knockdown. A system biology approach was adopted to investigate different BC gene expression datasets, dividing the patients by low or high KD score (corresponding to high or low TS, respectively). As a further confirmation of a strong association with EMT, a previously established signature of genes up-regulated during EMT [26] was found significantly enriched in patients with a low TS KD score (Fig. 5c). Moreover, a gene set predicting poor prognosis was found enriched in patients with low KD score (Fig. 5d), and similar associations with the grade of differentiation were found using both the TS KD score and TS expression to segregate the patients (Supplementary Fig. 4B, C). Survival analysis showed a strong prognostic impact of the TS-knockdown signature, indicating a poorer prognosis for the patients with a lower score or higher TS in all subtypes (Fig. 5e), and a trend for a significant impact in TNBC (Fig. 5f). The KD score was also significantly correlated with all the major histopathological variables in BC, including histology, grade of differentiation and size of the tumors (Fig. 5g–j). All these data together indicate that TS promotes EMT-driven aggressive BCs.

### TS enzymatic activity and thymidine catabolism are essential for maintaining BC de-differentiation

**Fig. 6:**
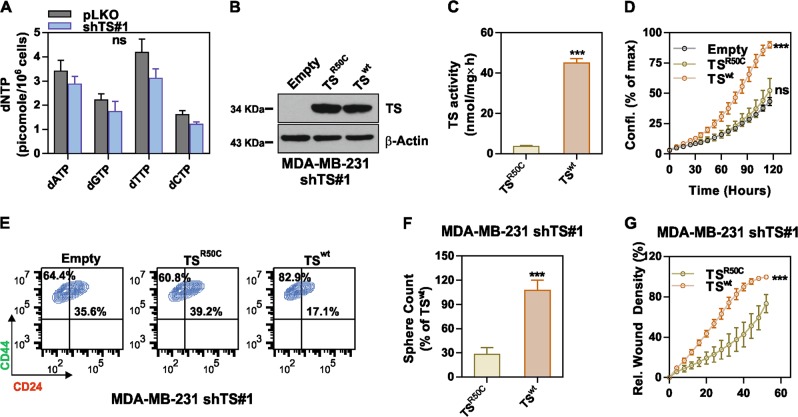
TS enzymatic activity is essential for maintenance of BC de-differentiation. **a** Quantification of deoxyribonucleotide triphosphate (dNTPs) in MDA-MB-231 cells with TS knockdown and control cells. **b** Reconstitution of enzymatically inactive (TS^R50C^) or wild-type (TS^wt^) TS in MDA-MB-231 shTS#1 cells. **c** Measurement of TS enzymatic activity in MDA-MB-231 shTS#1 cells expressing TS^R50C^ or TS^wt^ (*p* < 0.0001, unpaired two tailed *t*-test) (**d**) Proliferation (*p* = 0.0001, two-way ANOVA, Dunnett multiple comparison), **e** CD44/CD24 FACS profile, **f** sphere-forming ability (*p* < 0.0001, unpaired two tailed *t*-test), and **g** migration capacity (*p* < 0.0001, two-way ANOVA, Turkey’s multiple comparison) of cells that express either TS^R50C^ or TS^wt^. Experimental data are representative from at least two independent experiments with similar results. Points are avg ± SD

We then aimed at determining the impact of TS enzymatic activity on de-differentiation. TS is the only de novo source of thymidylate (dTMP), which is either further phosphorylated to maintain the dNTP pools for DNA synthesis, or directed to degradation via sequential phosphorolytic cleavages. MDA-MB-231 shTS#1 cells where subjected to dNTP quantification, but, consistently with the previously observed normal growth, no significant change in dTTP or other dNTPs was detected (Fig. 6a). This suggested that the differentiation observed in shTS cells was not caused by dNTP imbalance, as shown in other CSC models upon knockdown of specific NM-related enzymes [13], and that while a baseline TS level is required for the cells to grow, increased TS activity could be partly involved in other cellular functions. To functionally investigate the role of TS enzymatic activity, we reconstituted shTS#1 cells with either a wild type (TS^wt^) expressing silent mutation in shRNA binding region or an enzymatically inactive (TS^R50C^) form of TS [27]. Western blotting confirmed the successful transduction (Fig. 6b) and TS enzymatic activity assay confirmed the loss of catalytic activity in the TS^R50C^ overexpressing cells (Fig. 6c). Strikingly, MDA-MB-231 cells overexpressing the TS^wt^ enzyme increased proliferation (Fig. 6d), reduced the population of differentiated CD24^+^ cells (Fig. 6e), formed more mammospheres (Fig. 6f) and were significantly more migratory (Fig. 6g) compared to TS^R50C^ cells overexpressing the catalytically inactive TS.

**Fig. 7:**
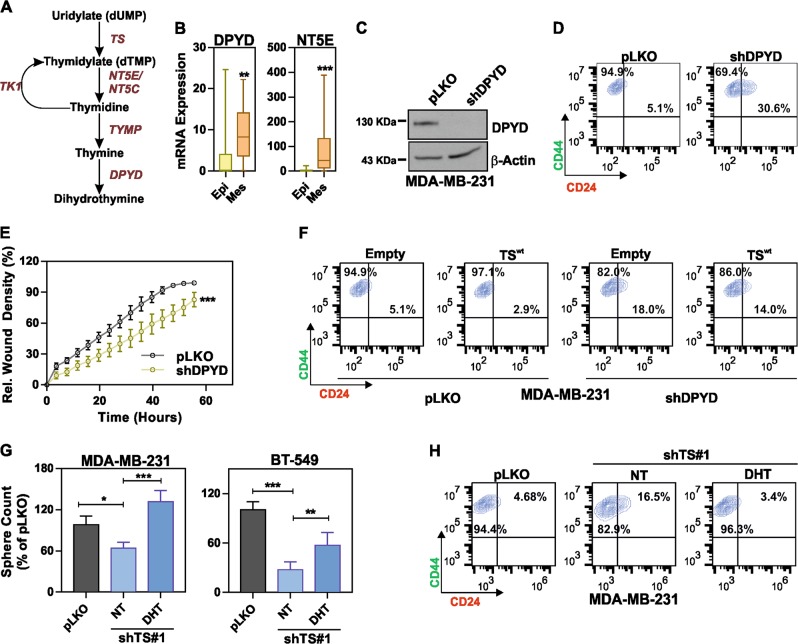
TS regulates de-differentiated tumor phenotype via DPYD-mediated thymidine catabolism. **a** Scheme of the thymidylate catabolic pathway. **b** DPYD (*p* = 0.0031) and NT5E (*p* = 0.0001) mRNA expression levels in epithelial and mesenchymal BC cell lines (CCLE dataset). The statistical tests are unpaired two tailed *t-*test. **c** Western blot quantification of DPYD knockdown in MDA-MB-231, and the effects on (**d**) CD44/CD24 profile and **e** migratory ability (*p* < 0.0001, two-way ANOVA, Sidak’s multiple comparison). **f** FACS plots of MDA-MB-231 cells with DPYD knockdown overexpressing TS or an empty vector and stained with CD44/CD24. **g** Sphere counts for shTS#1 cells in MDA-MB-231 (*p* < 0.0394 between pLKO and NT, *p* < 0.0001 between NT and DHT) and BT-549 (*p* < 0.0001 between pLKO and NT, *p* = 0.0023 between NT and DHT, one-way ANOVA, Turkey’s multiple comparison) after treatment with 1 µM dihydrothymine (DHT). NT (DMSO) is the non-treated control. **h** CD24/44 profile showing the effect of 10 µM DHT on CD24^+^ population in MDA-MB-231 cells with TS knock-down. Points are avg ± S

**Fig. 8:**
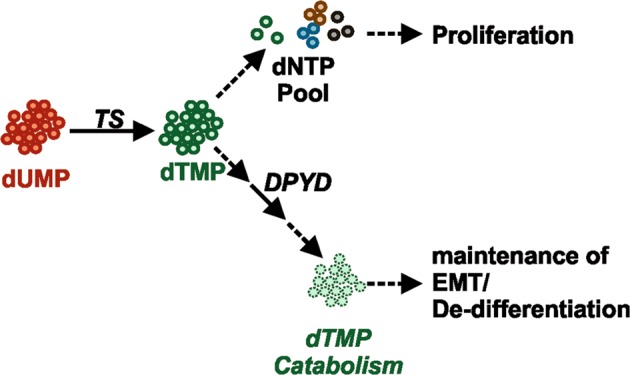
Schematic representation of TS mediated BC de-differentiation. Excessive thymidylate is fluxed in the catabolic pathway for degradation mediated by DPYD. DPYD catalyzed DHT maintains the de-differentiation in BC

These results clearly indicated that the TS enzymatic activity is essential for the maintenance of the EMT/CSCs phenotype. We therefore hypothesized that TS mediates de-differentiation via the nucleotide catabolism (Fig. 7a), rather than through the pathway leading to DNA synthesis. This was supported by the results of a recent shRNA screen on human immortalized breast cells, which revealed that pyrimidine catabolism mediated by dihydropyrimidine dehydrogenase (DPYD) was responsible for the EMT phenotype [28]. We therefore speculated existence of a TS-DPYD axis controlling EMT/CSCs in BC cells. Although DPYD mRNA expression did not show a significant prognostic role in BC patients (Supplementary Fig. 5A, B), analysis of expression data from the CCLE showed a marked increase in mesenchymal-like BC cells not only for DPYD, but also for the NT5E (CD73), which is one of the upstream 5′-nucleotidase responsible for catalyzing the first step of dTMP degradation (Fig. 7b). In order to functionally prove our hypothesis, we knocked down DPYD (Fig. 7c) and observed a significant increase in the population of CD24^+^ cells (Fig. 7d) and a loss of migratory ability (Fig. 7e), in line with what we observed in TS-deficient cells. However, the CD24^+^ enriching effect of DPYD knockdown could not be reverted by overexpressing TS (Fig. 7f), suggesting that TS enzymatic control of de-differentiation requires a DPYD-dependent pyrimidine catabolism. To further confirm it, we tested if the supplementation of DPYD enzymatic product DHT in medium could rescue the differentiation phenotype observed in TS knockdown cells. As a result, DHT supplementation significantly increased the counts of sphere (Fig. 7g) and reduced the CD24^+^ population (Fig. 7h) in TS knockdown cells, having no effect on the cell proliferation (Supplementary Fig. 5C). In summary, our data indicate that TS enzymatic activity and pyrimidine catabolism are essential for the maintenance of the BCSC phenotype. We therefore propose a model in which dTMP produced by TS-overexpressing cancer cells is not only metabolized to support the uncontrolled proliferation, but can also partially sustain de-differentiation and EMT via DPYD-based pyrimidines degradation (Fig. 8).

## Discussion

The aggressiveness and the de-differentiated phenotype of neoplastic cells have been strongly connected with alterations in specific metabolic pathways, especially those involved in the transformation of glucose [29–31], while the contribution of other pathways is still majorly unexplored. Elevation in NM is typically associated with the tumor cells’ increased demand for DNA precursors to sustain the uncontrolled proliferation [11]. However, a few studies have shown that some NM enzymes are functionally involved in de-differentiation processes, like the ecto-nucleotidase ENPP1 in the maintenance of the CSCs-like state in glioblastoma [13]. Similarly, treatment with non-toxic doses of TS-inhibiting drugs was found sufficient to induce a differentiation in multiple myeloma CSCs and sensitize the cells for radiotherapy [14]. These observations, together with the first demonstration of a connection between TS and EMT [17], prompted us to investigate the EMT-driven TNBC model [3, 22]. Functional experiments clearly indicates that loss of TS altered the de-differentiated phenotype of TNBCs, reducing CD44^+^CD24^−^ cells, and suppressing migratory and sphere-forming ability. Consistently, this was accompanied by a robust suppression of EMT-associated genes, as evaluated by RNA-seq analysis. In vivo, two independent animal models, confirmed by an in vitro extravasation assay, revealed an augmented propensity for intravasation and metastasis formation in cells with TS knockdown. These findings are in line with previous reports in which EMT-suppressing conditions enhanced the metastatic colonization capacity of mesenchymal-like BC cell lines [32, 33], and a plausible explanation comes from recent studies, which found that the tumor-initiating and metastatic potential is maximum when breast and other cancer cells are in an intermediate EMT (hybrid) state [34–38], leading to a partial revision of the initial model of EMT in cancer metastasis [35, 39, 40]. Our in vivo data therefore reflect the complicated nature of EMT, which may also vary in a context-dependent fashion [39, 41]. Targeting TS in other cells, in fact, exerted a clear anti-metastatic effect [42]. Nevertheless, both the immunohistochemical quantification of TS protein levels and the TS knockdown scores derived from the RNA-seq data were strongly associated with BC de-differentiation and prognosis, indicating a pivotal role of TS in the malignancy of BC. Interestingly, only a moderate co-expression between TS and the proliferation marker Ki67 was found, in line with previous data from other cancers [43, 44], supporting our model that TS activity could be implicated in other cellular functions independent of proliferation. We found, in fact, that the EMT/CSC suppression imposed by a level of TS knockdown, which did not perturb cells’ proliferation and dNTP balance was rescued by overexpressing wild-type TS, but not by a catalytically inactive mutant. This was particularly important because it ruled out the possible contribution of non-enzymatic activities of TS [45], directly pointing at the enzymatic activity as the EMT/CSC driving force. Since dihydropyrimidine accumulation was previously shown to control EMT in BC [28], we tested and demonstrated the hypothesis that high TS enzymatic activity in cancer cells sustains de-differentiation and EMT via a DPYD-dependent pyrimidine catabolism. Several follow-up studies are needed, for instance, to (1) test by IHC the significance of DPYD in BC, and to identify the mechanisms by which DHT controls EMT/CSC, to understand if the underlying pathways could represent novel valuable therapeutic targets; (2) address the impact of other dTMP-transforming enzymes (like NT5E/CD73) and salvage pathways on EMT; (3) identify the regulatory pathways upstream of the TS-DPYD axis; (4) test a similar function for TS in other malignancies; (5) decipher the contribution of TS dysregulation to cancer-relevant pathways other than EMT (like inflammation). In any case, our data assert that the classical role of TS as a mere proliferation marker needs to be revisited. For instance, the previously identified oncogenic and tumor-initiating role of TS [27, 46] could be explained with the direct/indirect control of de-differentiation and stemness. The present data add the control of de-differentiation to the list of functions of TS (together with proliferation and DNA repair) that can affect the prognosis of BC patients. More efforts will need to be dedicated to clarify how the cancer cells balance proliferation and differentiation, and to what exact extent other nucleotide metabolic genes are implicated in these processes.

TS is a well-established target of chemotherapy, being inhibited by drugs like 5-fluorouracil (5-FU) or by folate analogues [23], and its overexpression in tumors represents a major mechanism of chemo-resistance. From the translational point of view, our finding that TS levels can be significantly different among BC subtypes contradicts earlier works [47] and may be useful to improve the treatment strategies. In our study, TS was found higher in the aggressive BC and in high-grade tumors, in line with previous observations in other cancers [43, 44, 48], and this information could be clinically important for predicting the efficacy of anti-TS drugs. The present study, as well as other pivotal works [49], can therefore anticipate subtype-specific susceptibilities to anti-TS drugs in BC. However, these results also highlight the clinical importance of carefully assessing the potential impact of TS-inhibition on metastasis formation and, therefore, further in vivo work and retrospective studies are warranted.

## Supplementary information

Supplementary Figure 1

Supplementary Figure 2

Supplementary Figure 3

Supplementary Figure 4

Supplementary Figure 5

Supplementary Table 1

Supplementary Table 2

Supplementary Table 3

Supplementary Figure legends
