# Supplementary material for: Thymidylate synthase maintains the de-differentiated state of triple negative breast cancers
Source: Cell Death Differ. 2019 Feb 8;26(11):2223–36. doi: 10.1038/s41418-019-0289-6 (PMC6888897; doi:10.1038/s41418-019-0289-6)
Supplement: Supplementary file 6 — Supplementary Table 1 [file 41418_2019_289_MOESM6_ESM.pdf]

Supplementary Table - 1

SYMBOL	CDH1	VIM	ratio	TYMS
MCF7	218.67	1.67	0.007637	27.17
HCC1428	173	1.47	0.008497	35.04
BT-483	217.85	2.21	0.010145	8.42
MDA-MB-175-VII	196.32	2.22	0.011308	16.32
T-47D	125.64	1.48	0.01178	25.26
UACC-893	160	1.9	0.011875	50.97
KPL-1	168.68	2.8	0.016599	28.58
HCC202	129.66	2.9	0.022366	37.95
BT-20	182.34	4.2	0.023034	58.37
HCC1419	74.12	1.87	0.025229	35.84
HCC1937	108.31	3.08	0.028437	52.08
MDA-MB-415	117.31	3.62	0.030858	21.6
EFM-192A	144.81	6.37	0.043989	34.48
HCC1806	41.61	1.9	0.045662	34.03
EFM-19	92.39	4.61	0.049897	19.15
CAL-148	57.64	2.95	0.05118	33.66
DU4475	23.29	1.2	0.051524	44.27
CAL-85-1	162.55	10.86	0.06681	93.43
JIMT-1	53.86	3.68	0.068325	59.23
UACC-812	106.19	8.46	0.079669	26.11
HCC70	159.03	16.66	0.10476	34.24
ZR-75-1	87.26	9.18	0.105203	39.68
BT-474	161.19	16.99	0.105404	54.66
MDA-MB-453	9.76	1.07	0.109631	27.64
CAMA-1	21.21	2.37	0.11174	35.86
HCC1954	152.19	21.07	0.138445	29.55
HCC2218	8.58	1.63	0.189977	16.35
MDA-MB-468	27.59	12.47	0.451975	93.8
ZR-75-30	3.45	3.94	1.142029	17.42
MDA-MB-134-VI	5.31	6.12	1.152542	17.56
MDA-MB-361	210.5	250.84	1.191639	38.17
HCC1599	33.04	45.93	1.390133	59.18
HCC1187	27.52	40.91	1.486555	50.44
HCC38	45.74	98.84	2.160909	30.26
CAL-51	12.49	56.92	4.557246	78.29
HCC1143	79.47	506.06	6.367938	103.97
HCC2157	50.98	419.13	8.221459	32.03
SK-BR-3	0.18	2.89	16.05556	46.68
AU565	0.09	3.79	42.11111	42.98
CAL-120	8.64	1123.38	130.0208	100.53
BT-549	0.46	1219.59	2651.283	72.99
MDA-MB-231	0.38	1099.13	2892.447	99.91
MDA-MB-436	0.1	486.9	4869	32.4
HS-739.T	0.38	2685.45	7066.974	19.22
HS-742.T	0.4	3303.5	8258.75	11.2
MDA-MB-157	0.17	1612.7	9486.471	128.93

HS-578T	0.33	3737.13	11324.64	30.99
HCC-1359	0.24	2849.64	11873.5	127.45
HMC-1-8	0.09	1120	12444.44	69.43
HS-343.T	0.34	4752.57	13978.15	68.27
HS-281.T	0.3	4951.55	16505.17	44.33
HS-606.T	0.25	4142.49	16569.96	20.82
