# Supplementary material for: Thymidylate synthase maintains the de-differentiated state of triple negative breast cancers
Source: Cell Death Differ. 2019 Feb 8;26(11):2223–36. doi: 10.1038/s41418-019-0289-6 (PMC6888897; doi:10.1038/s41418-019-0289-6)
Supplement: Supplementary file 7 — Supplementary Table 2 [file 41418_2019_289_MOESM7_ESM.pdf]

Supplementary Table - 2

	n
**All**	120
**Age (mean, min-max)**	63, 26-92
**Grade I, II, III**	15,44,61
**HER2**	2
**Lum A**	23
**Lum B/ HER2 neg**	35
**Lum B/ HER2 pos**	13
**TN**	47
