# Supplementary material for: Thymidylate synthase maintains the de-differentiated state of triple negative breast cancers
Source: Cell Death Differ. 2019 Feb 8;26(11):2223–36. doi: 10.1038/s41418-019-0289-6 (PMC6888897; doi:10.1038/s41418-019-0289-6)
Supplement: Supplementary file 8 — Supplementary Table 3 [file 41418_2019_289_MOESM8_ESM.pdf]

Supplementary Table - 3

TS (TYMS) knockdown in MDA-MB-231

Genes down-regulated upon KD		Genes up-regulated upon KD	
ZNF300	IL11	SEMA3E	SEL1L3
DOCK3	ITGBL1	EPB41L4A	SHISA3
B3GNT7	SAA1	RFPL4A	PLD5
MAGEC2	LTBP2	FST	CLEC2B
CYP24A1	KYNU	POU3F2	TIAM1
LCN2	ZNF519	IFIT2	SMAD9
CST1	CLMN	STOM	FZD8
COL6A2	LIMS2	CCDC149	LOX
IL1A	SCN5A	CSRP2	LCP1
PLCB4	RCN3	F2RL2	COL1A1
TNFRSF1B	TYMS	CXCR4	CXCL11
IL1B	HIST1H2BC	BDNF	SDPR
EEF1A2	VNN1	APLN	OASL
PAPPA	DAPK1	TNFAIP3	HMGN5
PMEPA1	STC1	PTGS2	UCA1
NAP1L3	COL6A1	PCYOX1L	KCNQ3
HIST1H2BN	BIN1	ADAMTS1	GSPT2
FAM133A	CPM	PDK3	KRBOX1
ZNF542P	AFF3	SPANXC	ADGRL3
CST4	TUSC3	EXPH5	ATP1A3
VIPR1	KCNS3	PRRG4	RGS4
APCDD1L	C3	C14orf37	ESM1
TNFRSF11B	INHBA	ST6GAL1	BCL11A
TLR2	ICAM1	KITLG	NKX2-1
CFAP45	MAPK13	PPARGC1A	HS6ST3
SLFN13	ICAM2	NR2F1	SERPINB2
SAA2	COL27A1	CRISPLD2	ID4
LOC100506178	TOX2	CXADR	HSPA12A
LRIG1	TATDN1	IFIT1	SCEL
C15orf48	TRMT12	ARRDC4	ZNF716
ALDH1A3	VDR	PTN	FOXA1
TRIML2		CELF2	FABP5
KISS1		MMP1	IGFBP5
CDA		HOOK1	MCTP2
PDZK1IP1		PROS1	FGF13
COL18A1		NRK	SFTA3
CARD11		SLAMF7	COL1A2
HIST3H2A		CD24	FBXL7
SHISA2		USP32P2	TMPRSS15
TNFSF15		HCLS1	NCAM2
HIST1H3H		SAMSN1	PGM5
SERPINA1		PCDH7	EPB41L3
