# Supplementary material for: Thymidylate synthase maintains the de-differentiated state of triple negative breast cancers
Source: Cell Death Differ. 2019 Feb 8;26(11):2223–36. doi: 10.1038/s41418-019-0289-6 (PMC6888897; doi:10.1038/s41418-019-0289-6)
Supplement: Supplementary file 9 — Supplementary Figure legends [file 41418_2019_289_MOESM9_ESM.docx]

**SUPPLEMENTARY FIGURE LEGENDS**

**Supplementary Figure 1. TS correlates with Ki67 in breast cancer.** (*A*) Plot showing correlation between percent of TS and Ki67 positivity, as evaluated by immunohistochemistry on samples from BC patents. Correlation determined by Pearson’s coefficient (R). (*B*) Kapan-Meier curves showing the prognostic significance of TS expression in basal and grade-III differentiated breast tumors. P values are log-rank tests.

**Supplementary Figure 2. TS elimination leads to cell death.** (*A*) Complete ablation of TS with shRNA mediated knockdown (shTS#3) in MDA-MB-231 cells. (*B*) Real-time measurement of growth in MDA-MB-231 cells following complete TS ablation (scale bar represents 150µm).

**Supplementary Figure 3. TS controls the de-differentiated status of TNBC cells.** (*A*) Knockdown of TS in BT-549 cells. pLKO is non-targeting shRNA control. (*B*) Effect of TS knockdown on CD44/CD24 profile. Positive cells have been gated based on the unstained BT-549 cells. Effect of TS knockdown on (*C*) migration (p = 0.0010, two-way ANOVA, Sidak’s multiple comparison) and (*D*) sphere formation capability (P = 0.0129, unpaired two tailed t-test).

**Supplementary Figure 4. TS knockdown represses EMT and de-differentiation in breast cancer.** (*A*) qPCR validation of RNA-seq results in MDA-MB-231 shTS#2. GSEA of patients having (*B*) high and low TS expression and (*C*) low or high TS KD score with previously published gene signature for genes downregulated in grade-III vs grade-I tumors.

**Supplementary Figure 5.** (*A*) mRNA expression levels of DPYD in breast tumor samples compared to the normal tissues. (*B*) Effect of DPYD expression on the overall survival of breast cancer patients (GSE45827). P-values are log-rank test. (*C*) Effect of 1µM dihydrothymine (DHT) on the proliferation in MDA-MB-231 cells with TS knock-down (one way ANOVA, Sidak’s multiple comparison).

**SUPPLEMENTARY TABLE LEGENDS**

**Supplementary Table 1.** Characteristics of the n=120 BC patients evaluated at IHC.

**Supplementary Table 2.** Gene expression levels of E-Cadherin (CDH1), Vimentin (VIM) and TS (TYMS) in BC cell lines belonging to the CCLE dataset.

**Supplementary Table 3.** Genes up- and down-regulated in MDA-MB-231 cells upon TS knockdown.
